# GHR is involved in gastric cell growth and apoptosis via PI3K/AKT signalling

**DOI:** 10.1111/jcmm.16160

**Published:** 2021-01-25

**Authors:** Hong‐Zhu Yan, Hua‐Feng Wang, Yueling Yin, Jue Zou, Feng Xiao, Li‐Na Yi, Ying He, Bo‐Sheng He

**Affiliations:** ^1^ Department of Pathology Seventh People's Hospital of Shanghai University of TCM Shanghai China; ^2^ Department of Pathology Ruijin Hospital, Shanghai Jiao Tong University School of Medicine Shanghai China; ^3^ Department of Pathology Haiyang People's Hospital Haiyang China; ^4^ Department of Ultrasound The Tumor Hospital of Nantong University Nantong China; ^5^ Department of Radiology Affiliated Hospital 2 of Nantong University Nantong China; ^6^ Clinical Medicine Research Center Affiliated Hospital 2 of Nantong University Nantong China

**Keywords:** cell cycle, cell growth and apoptosis, gastric cancer, GHR, PI3K/AKT signalling

## Abstract

Growth hormone receptor (GHR), the cognate receptor of growth hormone (GH), is a membrane bound receptor that belongs to the class I cytokine receptor superfamily. GH binding GHR induces cell differentiation and maturation, initiates the anabolism inside the cells and promotes cell proliferation. Recently, GHR has been reported to be associated with various types of cancer. However, the underlying mechanism of GHR in gastric cancer has not been defined. Our results showed that silence of GHR inhibited the growth of SGC‐7901 and MGC‐803 cells, and tumour development in mouse xenograft model. Flow cytometry showed that GHR knockout significantly stimulated gastric cancer cell apoptosis and caused G1 cell cycle arrest, which was also verified by Western blot that GHR deficiency induced the protein level of cleaved‐PARP, a valuable marker of apoptosis. In addition, GHR deficiency inhibited the activation of PI3K/AKT signalling pathway. On the basis of the results, that GHR regulates gastric cancer cell growth and apoptosis through controlling G1 cell cycle progression via mediating PI3K/AKT signalling pathway. These findings provide a novel understanding for the role of GHR in gastric cancer.

## 1 INTRODUCTION

Growth hormone (GH), a type of monopeptide strain hormone, is synthesized and secreted from anterior pituitary.^1^, ^2^ In the past decade, GH has gained extensive attention in tumour development, progression and metastasis, including breast cancer,^2^ prostate cancer,^3^ endometrial cancer,^4^ hepatocellular carcinoma ^5^ and gastric cancer.^6^ The relationship between GH and cancer has been widely reported in human and animal models.^7^, ^8^ Mice with GH transgene increased the incidence of spontaneous and carcinogen‐induced the development of hepatocellular carcinoma.^9^ Rather, mice with GH deficiency attenuated the susceptibility to carcinogen‐caused hepatocarcinogenesis.^10^ In addition, GH is reported to predict the poor survival outcomes for breast cancer and endometrial cancer patients.^2^, ^8^ Insulin‐like growth factor (IGF) signalling pathway is one of the important pathways involved in GH‐associated cancer progression. IGF‐1, a mitogen for both normal and neoplastic cells, exerts potent antiapoptotic and mitogenic activity in all cells and is expressed in many different types of cancer cells.^11^ GH and IGF‐1 are both required for cell differentiation and growth that GH serves to commit a precursor cell to a specific pathway of differentiation, and IGF‐1 to raise growth and replication.^12^ In cellular and animal models, GH and IGF‐1 promote tumour growth and progression.^11^ The reduced production of GH and IGF‐1 in animals resists to carcinogenesis, conversely, mice transgenic for human GH have increased rates of tumours.^11^, ^13^

Growth hormone receptor (GHR), the cognate receptor of GH, is a membrane bound receptor that belongs to the class I cytokine receptor superfamily.^14^ GHR gene is located on the fifth chromosome and is highly expressed in many types of human cancers, such as colorectal carcinoma and breast cancer.^2^ GH combines GHR with 191 amino acid monomeric peptides that is secreted from the eosinocyte anterior pituitary, inducing cell differentiation and maturation, initiating the anabolism inside the cells and promoting cell proliferation.^15^

It has been reported that there is a close relationship between growth factor and gastric mucosal disease.^16^ In the stomach, GHR is mainly distributed among parietal and chief cells, and GH plays roles in target tissues by binding to GHR.^6^ GH promotes water and electrolyte transport and calcium absorption in the gastrointestinal tract ^1^ and restores the intestinal and gastric mucosal weight in rats having a hypophysectomy.^17^ GHR expresses in isolated glands, gastric cell fractions and intestinal mucosa lineages.^17^ In addition, GH is associated with gastric cancer. GHR transcripts exist though out cancerous progression of gastric mucosa from adenomas to gastrointestinal cancer at various stages of growth and differentiation.^12^ GHR expression is significantly higher in primary gastric adenocarcinoma compared with normal gastric mucosa and is in connection with tumour stage and tumour differentiation.^6^

So far, little studies comprehensively and systematically detect the roles of GHR in gastric cancer. Therefore, to fill the gap, this study addressed the influences of GHR expression on cell growth and apoptosis of gastric cancer cell lines as well as tumour growth of mice model. Furthermore, we also investigated the mechanism underlying the association between GHR and gastric cancer. Our data suggest that GHR regulates gastric cancer cell growth and apoptosis through controlling G1 cell cycle progression via mediating PI3K/AKT signalling pathway, which provides a novel understanding for the role of GHR in gastric cancer.

## 2 MATERIALS AND METHODS

### 2.1 Patients and specimens

This study included 11 specimens of gastric cancer from patients who underwent surgery at Seventh People's Hospital of Shanghai University of TCM. All patients signed the informed consent. Tumours and normal mucosa were obtained in all cases. Tissues were frozen in liquid nitrogen and stored at −80°C.

### 2.2 Cell lines and antibodies

The human gastric cancer cell lines, including SGC‐7901, MGC‐803, HGC‐27 and BGC‐823, and two human gastric epithelial cell lines, such as GES‐1 and RGM‐1, were obtained from the American Type Culture Collection (ATCC). All cells were maintained in RPMI 1640 (GIBCO BRL, Grand Island, NY), supplemented with 10% FBS (GIBCO BRL, Grand Island, NY) and cultured at 37°C in 5% CO_2_ incubator. The antibodies were purchased from Sigma (β‐actin), Proteintech (GHR) and Cell Signaling Technology (PCNA, p‐PI3K‐Tyr458, P‐AKT‐Ser473, PI3K, AKT, CDK4, Cyclin D1, Cleaved‐PARP, IR DyeR 680 goat anti‐Mouse, IR DyeR 800 goat anti‐Mouse).

### 2.3 Animals

Five‐ to 6‐week‐old nude mice were obtained from Shanghai SLAC Laboratory Animal Co, and were used for tumour development. All mice were cultured in a sterile environment and daily 12‐h light/12‐h dark cycle.

### 2.4 Cell transfection and ELISA Kit detection

SGC‐7901 and MGC‐803 cells were transfected with siRNA plasmids of GHR by using Lipofectamine 2000 transfection reagent (Life Technologies, Grand Island, NY) in accordance with the manufacturer's instructions. The two siRNAs target GHR were 5‐GCAACCAGAUCCACCCAUUTT‐3 and 5‐GCACCACGCAAUGCAGAUATT‐3. Six‐well plates were used to culture the two gastric cancer cell lines overnight, and then, 5 μg plasmids were added into cells mixed with lipofectamine solution. At last, the transfection efficiency was detected by Western blot. We detected the expression of GH by ELISA Kit for Growth Hormone (Cloud‐Clone Corp. No. SEA044Bo).

### 2.5 Western blot assay

Lysis buffer was utilized to extract the proteins of cells, which were then separated on 10% SDS‐PAGE gels, and transferred from gels to polyvinylidene difluoride (PVDF) membranes. And PVDF membranes were blocked with 5% BSA, following by incubation with primary antibodies overnight at 4°C, and incubation with diluted goat polyclonal anti‐rabbit IgG secondary antibody (1:2000; Abcam). At last, an enhanced chemiluminescence system was used to evaluate protein levels.

### 2.6 Quantitative real‐time PCR (qRT‐PCR)

Total RNA was isolated by using TRIzol reagent (Invitrogen, Carlsbad, CA), and reverse transcribed to cDNA using a First‐Strand cDNA Synthesis kit (TaKaRa Bio, Kusatsu, Japan). QRT‐PCR reactions were performed with the TaqMan Real‐Time PCR Master Mix (Thermo Fisher Scientific), using GHR primers. Samples were run in triplicates. The relative expression level of GHR in each type of cell was analysed by using 2^−ΔΔCt^ method.

### 2.7 Colony formation assay

The clonogenic ability of cells was assessed by performing colony formation assay in anchorage‐independent growth conditions. In detail, after gastric cancer cells being transfected with siGHR or siRNA, cells were seeded in 60‐mm dishes containing a top layer of 0.7% agar and a bottom layer of 1% agar. Plates were incubated at 37°C for 4 weeks and then stained with 0.2% crystal violet.

### 2.8 MTT assay

Cell viability was measured by using a colorimetric 3‐(4,5‐dimethylthiazol‐2‐yl)‐2,5‐diphenyl‐tetrazolium bromide (MTT) assay (Roche, Indianapolis, IN, USA). After gastric cancer cells being transfected with siGHR or siRNA, cells were cultured for 24, 48, 72 or 96 hours, and MTT solution was added to each well for 4 hours at 37°C. And then, DMSO was added to solubilize MTT formazan crystals, and the optical density was determined at 570 nm (OD570) using a ELISA plate reader (Model 550; Bio‐Rad, USA).

### 2.9 Cell apoptosis assay

Cell apoptosis was evaluated by flow cytometry analysis using an annexin‐V FITC Apoptosis Detection Kit I (BD Biosciences, San Jose, CA). Briefly, after gastric cancer cells being transfected with siGHR or siRNA for 48 hours, cells were stained with annexin V‐fluorescein isothiocyanate (FITC) and propidium iodide (PI). And then, flow cytometry was conducted on a FACScan flow cytometer (Becton Dickinson, San Jose, CA).

### 2.10 Cell cycle analysis

DNA flow cytometry analysis was performed to determine cell cycle. Briefly, after gastric cancer cells being transfected with siGHR or siRNA for 48 hours, cells were fixed in 70% ethanol at 4°C and treated with RNase. And then, cells were stained with PI in the dark for 30 minutes. Finally, samples were analysed by FACScan flow cytometer (Becton Dickinson, San Jose, CA).

### 2.11 Tumour xenografts

Ten 4‐ to 6‐week‐old female BALB/c nude mice were used, and they were randomized into two groups. A total of 2 × 10^6^ MGC‐803 cells transfected with siGHR or siRNA were injected into the flanks of nude mice. Tumour volumes were measured each 3 days by using a digital caliper. Mice were sacrificed after 24 days, and tumour weight was measured. And then, tumours were excised for paraffin block preservation.

### 2.12 Statistical analysis

All experiments were repeated at least three times to show the statistical significance. The data are expressed as the mean ± standard error of the mean (SEM). Statistical analysis was performed between the groups of data by paired Student's *t* test. *P* values of <0.05 were considered as statistically significant.

## 3 RESULTS

### 3.1 GHR is highly expressed in gastric cancer

**FIGURE 1:**
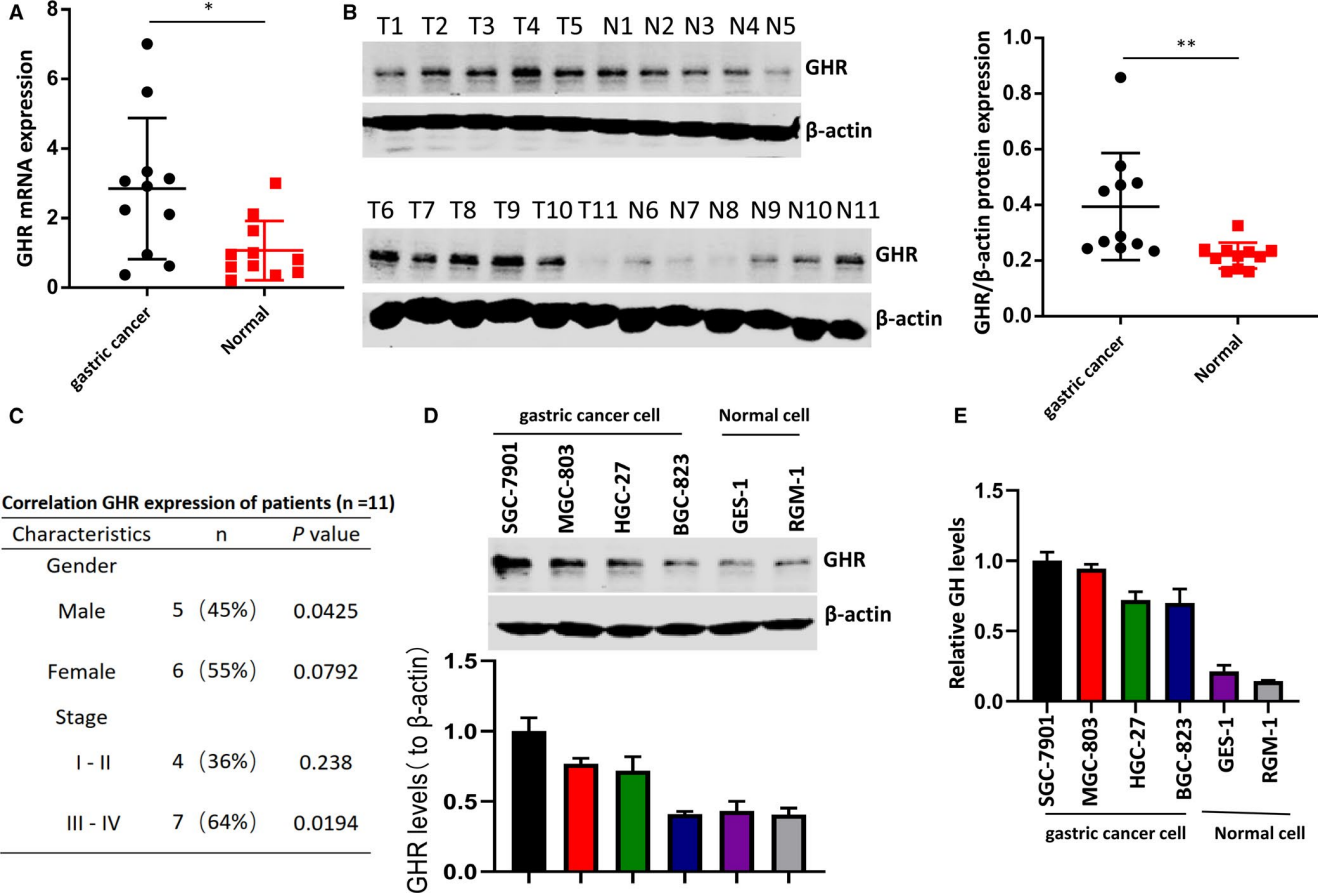
The expression levels of GHR in gastric cancer tissues and cell lines. A, GHR was highly expressed in tumour tissues compared with normal mucosa tissues. B, The expression of GHR was examined by Western blotting in tumour tissues compared with normal mucosa tissues. C, The clinicopathologic characteristics of patients and the correlation of GHR were shown. P value was determined by Student's *t* test. D, GHR level was significantly increased in gastric cancer cell lines (SGC‐7901, MGC‐803, HGC‐27 and BGC‐823) compared with human gastric epithelial cell lines (GES‐1 and RGM‐1). *P < 0.05 as determined by Student's t test. E, The expression of GH in four gastric cancer cell lines and two human gastric epithelial cell line was determined by ELISA

Total 11 tumour tissues and 11 normal mucosa samples from gastric cancer patients were used to detect the expression level of GHR. The results showed that GHR was highly expressed in tumour tissues compared with in adjacent normal mucosa samples (Figure 1A, Figure S1A). The protein level of GHR in gastric cancer cell was assessed by western blot (Figure 1B). In addition, the clinicopathologic characteristics of patients and the correlation of GHR demonstrated that GHR was correlated to late‐stage and male patients (Figure 1C). Next, four gastric cancer cell lines, including SGC‐7901, MGC‐803, HGC‐27 and BGC‐823, and two human gastric epithelial cell lines, GES‐1 and RGM‐1, were used. We showed that increased GHR productions were observed in gastric cancer cells compared with normal cells (Figure 1D), suggesting GHR expression might play a role in gastric cancer development. Interestingly, the level of growth hormone (GH) in gastric cancer cells was high compared that that in human gastric epithelial cells (Figure 1E).

### 3.2 GHR deficiency caused the reduction of gastric cancer cell growth

**FIGURE 2:**
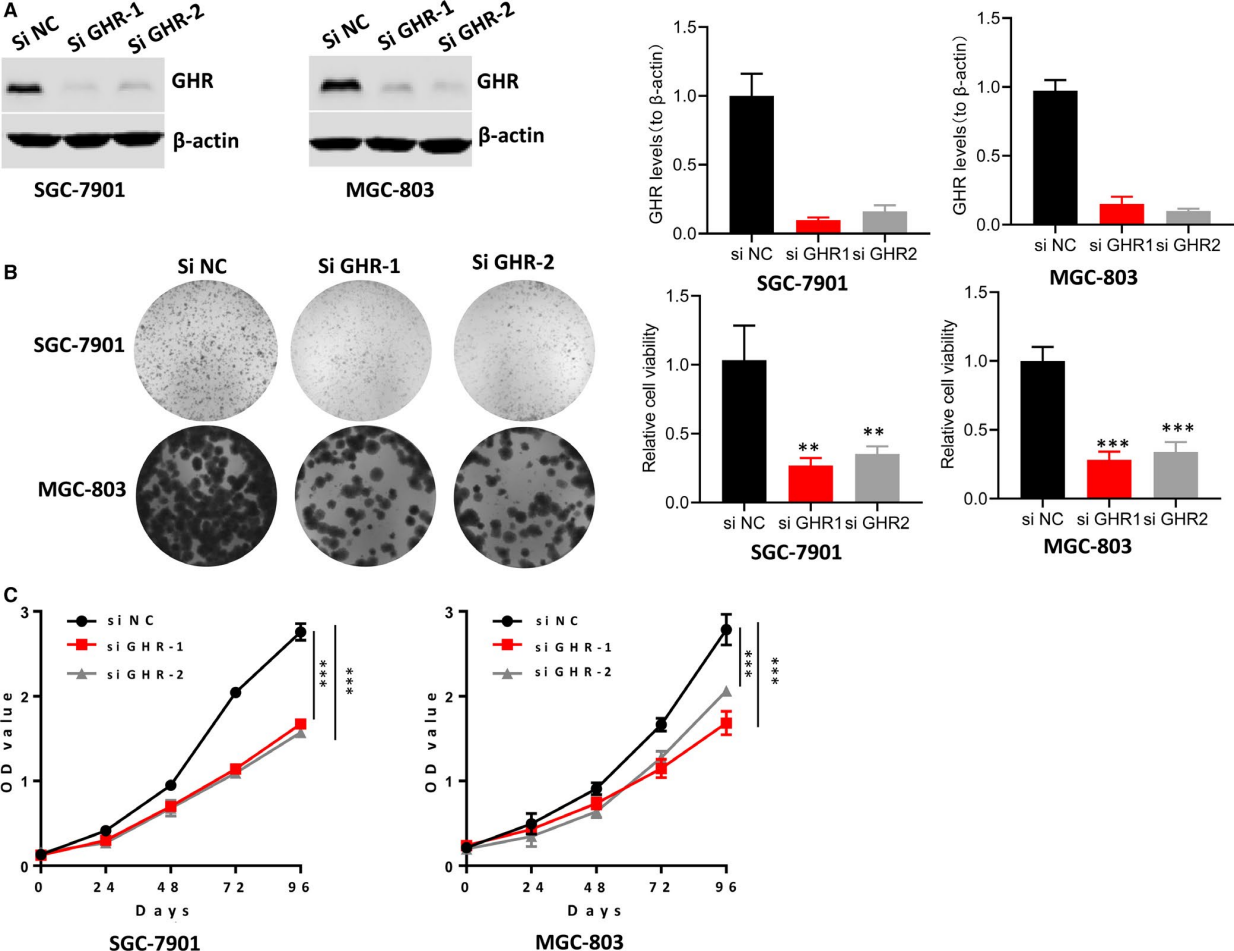
The impact of GHR on the growth of gastric cancer cell. A, GHR expression was significantly decreased in SGC‐7901 and MGC‐803 cells with siGHR transfection. B,C: Silencing GHR significantly inhibited SGC‐7901 and MGC‐803 cells proliferation. ***P < 0.001

To evaluate the effect of GHR on gastric cancer cell growth, SGC‐7901 and MGC‐803 cells were transfected with siGHR‐1 or siGHR‐2. The efficiency of transfection was detected by Western blot (Figure 2A). Colony formation assay showed that silence of GHR decreased the growth of SGC‐7901 and MGC‐803 cells (Figure 2B). MTT assay showed the same results that the OD value of SGC‐7901 and MGC‐803 cells with GHR deficiency was markedly reduced compared with the control group (Figure 2C).

### 3.3 GHR was associated with gastric cancer cell apoptosis and cell cycle

**FIGURE 3:**
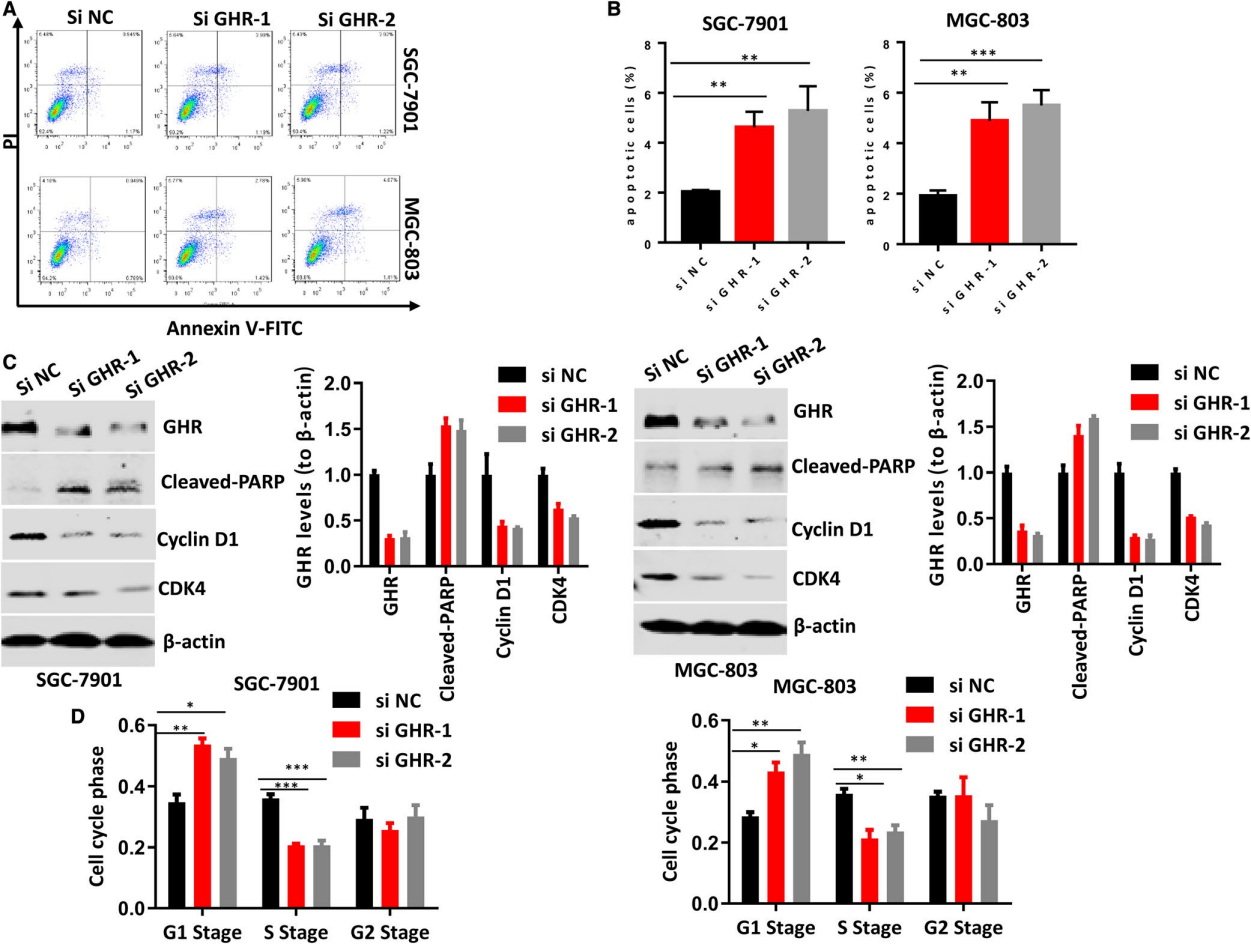
The roles of GHR in the apoptosis of gastric cancer cell and cell cycle. A,B: The reduction of GHR significantly induced the apoptosis of SGC‐7901 and MGC‐803 cells. C, GHR deficiency stimulated the protein levels of cleaved‐PARP and decreased the protein levels of CDK4 and Cyclin D1 in SGC‐7901 and MGC‐803 cells. D, GHR inhibition caused G1 cell cycle arrest. *P < 0.05. **P < 0.01. ***P < 0.001. Multi‐way ANOVA was used to analyse the data

Our results revealed that GHR silence decreased gastric cancer cell growth; however, the cause of this reduction is unclear. Thus, we further investigate the role of GHR in cell apoptosis and cell cycle of SGC‐7901 and MGC‐803 cells. Flow cytometry showed that GHR knockout significantly stimulated gastric cancer cell apoptosis compared with control group (Figure 3A,B). In addition, inhibiting GHR induced the protein level of cleaved‐PARP in SGC‐7901 and MGC‐803 cells (Figure 3C). The poly‐ADP‐ribose polymerases (PARP) family contributes to both DNA repair and cell apoptosis.^18^ Cleavage of PARP is a major substrate of caspase‐3 and caspase‐7, and functions as a valuable marker of apoptosis.^19^ Importantly, cell cycle regulators cyclin D1 and CDK4 were decreased in GHR silenced cells (Figure 3C), indicating the GHR was involved in cell cycle arrest. In response to DNA damage and replication blocks, cells may prevent cell cycle progression.^20^ Thus, we detected the impact of GHR inhibition on gastric cancer cell cycle progression by cytofluorimetry. GHR deficiency stimulated SGC‐7901 and MGC‐803 cells accumulation in G1 stage, and cell growth was then significantly decreased in S stage (Figure 3D). However, in G2 stage, there were no significantly differences between cells transfected with siGHR and control group. These results suggested that inhibiting GHR prevented gastric cancer cell progression from G1 to S stage. This might be the reason for cell growth reduction and cell apoptosis increase that caused by GHR blockage.

### 3.4 GHR regulated PI3K/AKT signalling pathway in gastric cancer cells

**FIGURE 4:**
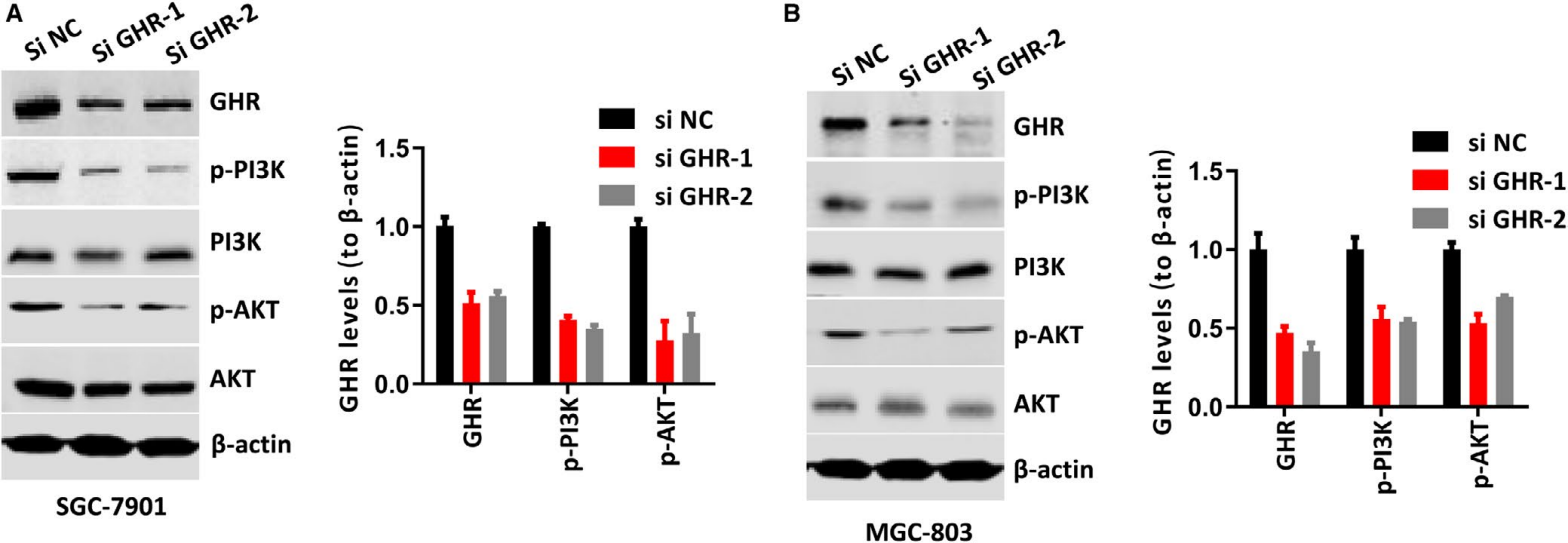
GHR regulated the activation of PI3K/AKT signalling pathway. A,B: Inhibition of GHR significantly reduced the protein levels of p‐PI3K and p‐AKT in both SGC‐7901 and MGC‐803 cells

In order to evaluate the mechanism underlying the role of GHR in gastric cancer progression, this study examined the association between GHR and PI3K/AKT signalling pathway. Our data showed that GHR silence inhibited the protein levels of p‐PI3K and p‐AKT in both SGC‐7901 and MGC‐803 cells, suggesting GHR was involved in gastric cancer by regulating PI3K/AKT signalling pathway (Figure 4).

### 3.5 GHR silence inhibited tumour growth of xenograft mouse model

**FIGURE 5:**
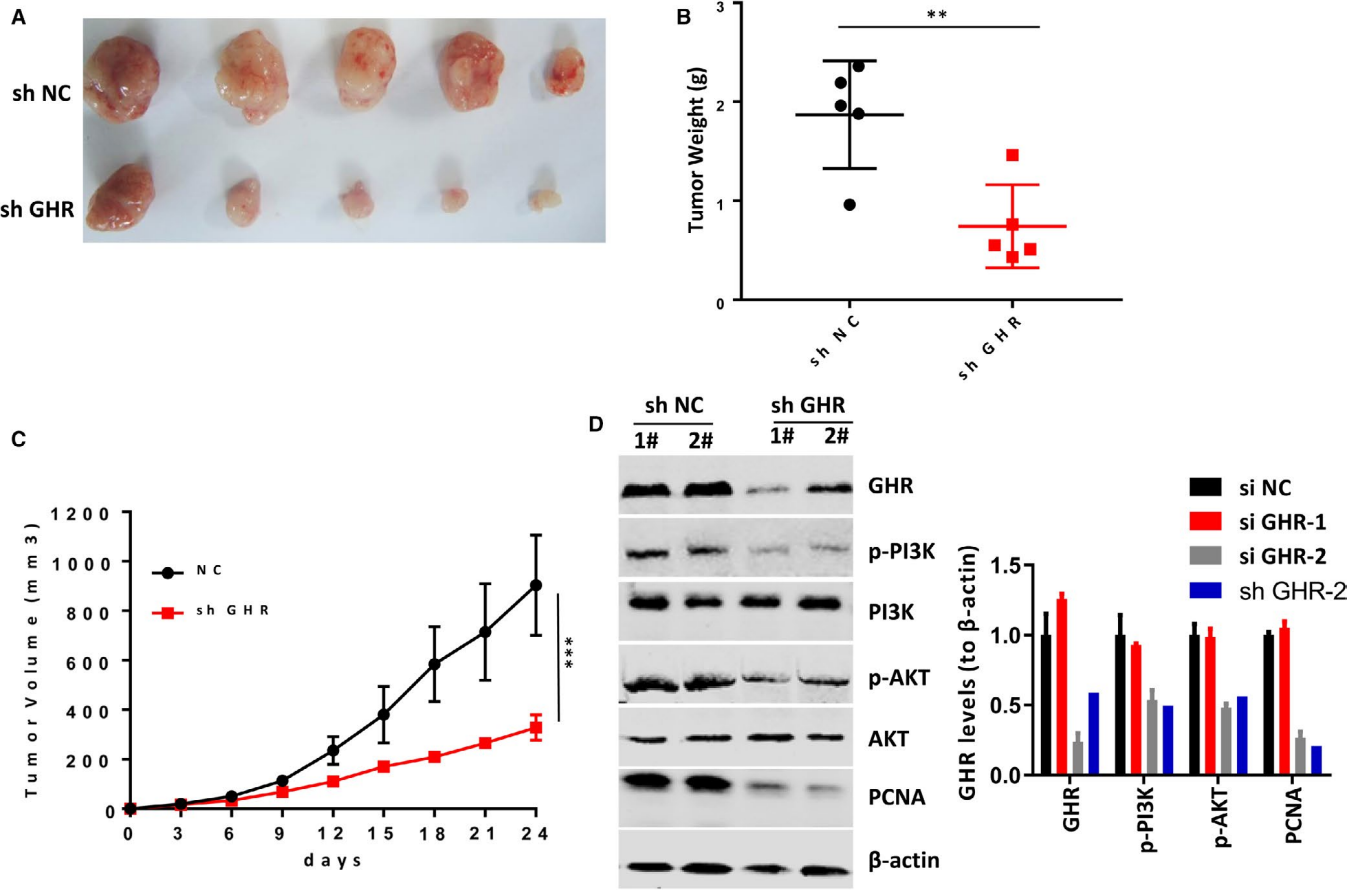
GHR reduction inhibited tumour growth in the xenograft mice model. A, Tumour size was smaller in mice established by MGC‐803 cell with shGHR transfection than that established by cells with shRNA. B, GHR reduction reduced tumour weight. C, GHR reduction caused tumour volume decrease. **P < 0.01. ***P < 0.001. D, The protein expression of GHR, PCNA, PI3K and AKT in xenograft tumours was detected by Western blotting

Gastric cancer cell was injected into mice to establish xenograft mouse model. Our results showed that tumour weight was significantly smaller in mouse models with GHR deficiency than that in control group (Figure 5A,B). In addition, tumour volume was also detected each three days. After 24 days, tumour volume was remarkably inhibited by GHR blockage in mouse models (Figure 5C). Furthermore, we detected the GHR, PCNA (one tumour growth marker) and PI3K/AKT levels in tumours derived from cells silenced GHR and found the expression of GHR still decreased in the dissected tumours (Figure 5D). In summary, these results suggested that GHR silence inhibited tumour growth in animal model.

## 4 DISCUSSION

Our study focused on the possible relationship between gastric cancer and GHR. We found that GHR was highly expressed in gastric cancer cell lines, and inhibiting its expression caused cell growth suppression and cell apoptosis increase during G1‐S transition. The prevention of cell cycle progression from G1 to S stage transition might result from the inhibiting effect of GHR deficiency on PI3K/AKT signalling pathway. In addition, xenograft mouse model experiment showed GHR deficiency inhibited tumour growth.

Growth hormone plays a critical role in normal linear growth and regulates metabolic progresses^21^, ^22^ and has been widely used clinically that it was approved by the U.S Food and Drug Administration (FDA) in the treatment of GH deficiency in adults and human immunodeficiency virus wasting syndrome.^23^, ^24^, ^25^ In addition, GHR has been reported to be involved in various types of cancer in recent years, including gastric cancer. Gastric cancer is the fourth most common cancer in incidence and the second most frequent in mortality among all cancers worldwide.^26^ Its 5‐year survival rate is less than 25%.^27^ Yang et al found that GHR was expressed in human primary gastric cancer, and the expression level was higher in tumours than in normal mucosa.^6^ In our study, GHR was highly expressed in gastric cancer tissues and cell lines. However, the roles of GHR in gastric cancer are still unclear. Thus, we further investigated the effects of GHR on gastric cancer cell growth and apoptosis. Colony formation assay and MTT assay both showed that silence of GHR decreased the growth of SGC‐7901 and MGC‐803 cells. Mouse xenograft model of human gastric cancer cell, which was transfected with siGHR showed the same result that silencing the expression of GHR in cell inhibited tumour development and growth. Flow cytometry showed that GHR knockout significantly stimulated gastric cancer cell apoptosis compared with control group, which was also verified by Western blot that GHR deficiency induced the protein level of cleaved‐PARP, a valuable marker of apoptosis.^19^ Interestingly, this study further found that inhibiting GHR prevented cell cycle from G1 to S stage transition. Previous studies have reported that oncogenic processes play their greatest role by targeting particular regulators of G1 phase progression.^28^ Unlike transition from S, G2 and M phases, G1 progression normally dependents on the induction of mitogens, which can be blocked by antiproliferative cytokine.^9^ The hallmark of cancer is uncontrolled cell proliferation, and cancer cell typically damages genes that directly regulate their cell cycle. GHR deficiency inhibited gastric cancer cell growth and induced cell apoptosis, further blocking cell cycle progression from G1 to S phase.

GHR has been reported to activate signalling transduction pathways that are critical for cell growth and survival. GH binds to GHR and facilitates increased binding of JAK2, which further influences the signal transducers and activators of transcription (STAT) signalling pathway, insulin‐like growth factor (IGF) signalling pathway, insulin receptor substrate proteins involved in the phosphoinositide 3‐kinase/AKT (PI3K/AKT) signalling pathway, and the mitogen‐activated protein kinase (MAPK) signalling pathway.^2^ In gastric cancer, the effect of GHR on tumour has been reported to involve PAK1‐STAT3/NF‐κB signalling^26^ and IGF signalling pathway.^6^ In this study, a reduction in the phosphorylation of PI3K and AKT pointed that GHR deficiency inhibited the PI3K/AKT signalling pathway in gastric cancer cells. PI3K/AKT signalling is one of the most important intracellular pathways that is frequently activated in diverse cancers, which regulates cell proliferation, differentiation, cellular metabolism, apoptosis and cancer cell survival.^29^ Previous studies have demonstrated that PI3K inhibitor, LY294002 induces G1 cell cycle arrest in melanoma, osteosarcoma, prostate cancer and ovarian cancer cells.^30^, ^31^ Cell cycle progression through the G1‐S transition is considered to be controlled by the activity of G1 phase cyclins and CDKs, including cyclin D1, CDK4 and CDK6, which stimulate G1 cell cycle progression.^30^ In cancer cells, inhibition of PI3K is reported to markedly suppress the expression levels of cyclin D1, CDK4 and the phosphorylation of Rb at Ser780, Ser795 and Ser807/811.^30^, ^31^ GHR silence inhibited PI3K/AKT signalling pathway in this study, which might suppress the activation of cyclins and CDKs, further leading to the prevention of cell cycle progression through the G1‐S transition.

## 5 CONCLUSION

In the present study, we provide a novel understanding for GHR in gastric cancer that GHR regulates gastric cancer cell growth and apoptosis through controlling G1 cell cycle progression via mediating PI3K/AKT signalling pathway.

## AUTHOR CONTRIBUTION

**Hongzhu Yan:** Conceptualization (equal); Investigation (equal). **Huafeng Wang :** Investigation (equal). **Yueling Yin:** Investigation (equal). **Jue Zou:** Investigation (equal). **Feng Xiao:** Investigation (equal). **Lina Yi:** Investigation (equal). **Ying He:** Investigation (equal). **Bo Sheng He:** Conceptualization (equal); Writing‐original draft (equal); Writing‐review & editing (equal).

## Supporting information

Fig S1
