# Supplementary material for: GHR is involved in gastric cell growth and apoptosis via PI3K/AKT signalling
Source: J Cell Mol Med. 2021 Jan 25;25(5):2450–8. doi: 10.1111/jcmm.16160 (PMC7933969; doi:10.1111/jcmm.16160)
Supplement: Supplementary file 1 — Fig S1 [file JCMM-25-2450-s001.pdf]

Supporting figure 1 The expression levels of GHR in clinical gastric cancer tissues

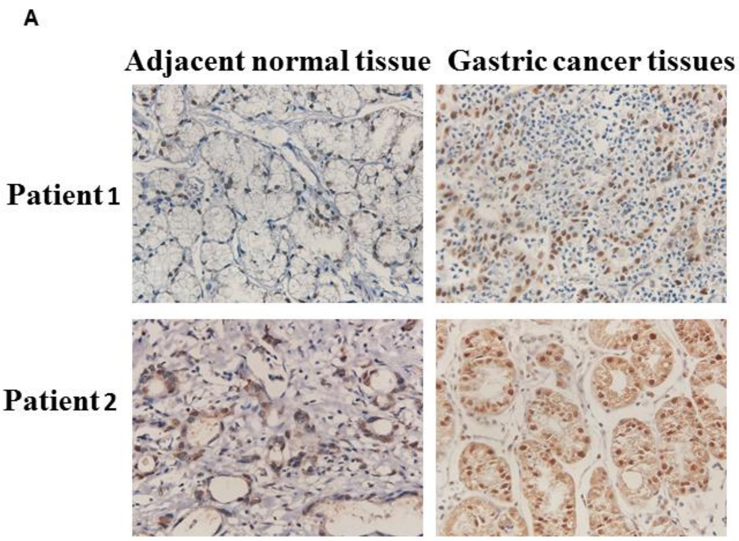

Figure legends

Supporting Figure 1: The expression levels of GHR in clinical gastric cancer tissues

A. GHR was highly expressed in tumor tissues compared with adjacent normal tumor tissues.
